# Supplementary material for: Real‐Time Self‐Optimization of Quantum Dot Laser Emissions During Machine Learning‐Assisted Epitaxy
Source: Adv Sci (Weinh). 2025 May 2;12(27):2503059. doi: 10.1002/advs.202503059 (PMC12279205; doi:10.1002/advs.202503059)
Supplement: Supplementary file 1 — Supporting Information [file ADVS-12-2503059-s001.docx]

**Supporting Information**

Supporting Information is available from the Wiley Online Library or from the author.

This work presents a machine learning-driven approach integrated with in-situ high-energy electron diffraction (RHEED) monitoring to automate the optimization of quantum dot (QD) growth for lasers. We achieve a 3.2-fold increase in photoluminescence intensity alongside a narrow spectral linewidth. The QD lasers fabricated demonstrate efficient lasing at 1240 nm with low threshold currents, highlighting a scalable alternative to manual optimization.

Real-time Self-optimization of Quantum Dot Laser Emissions During Machine Learning-Assisted Epitaxy


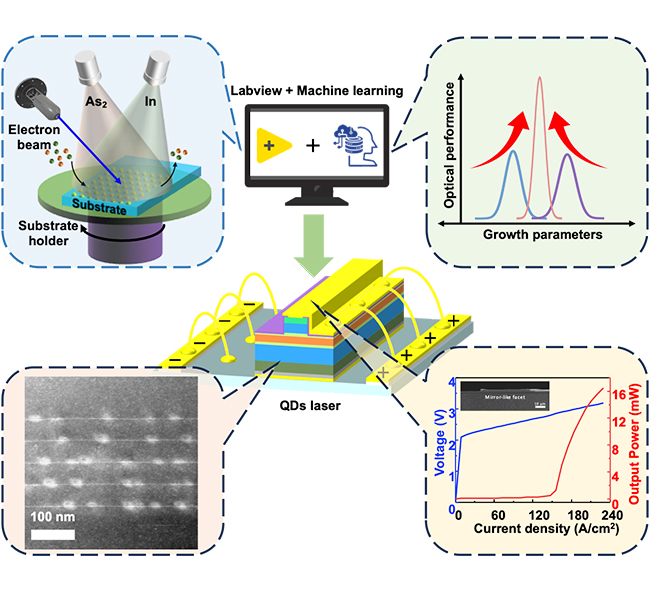


Supporting Information

Real-time Self-optimization of Quantum Dot Laser Emissions During Machine Learning-Assisted Epitaxy

Chao Shen, Wenkang Zhan, Shujie Pan, Hongyue Hao, Ning Zhuo, Kaiyao Xin, Hui Cong, Chi Xu, Bo Xu, Tien Khee Ng, Siming Chen, Chunlai Xue, Zhanguo Wang, and Chao Zhao*

1. **The PL result of InAs QDs growth with different V/III ratios**

We cut a 2-inch n-GaAs wafer into 4 parts. We then grew 4 InAs/GaAs QD samples on these slices, each with a different V/III ratio. The PL of the 4 samples were obtained with a laser power density of 423.25 W/cm², a higher excitation than others due to their weak emission. The results in **Figure S1** show that the intensity is highest at a V/III ratio of 120. Upon analyzing the intensities and full width at half-maximum (FWHM) of these 4 samples, we observed that the PL intensities initially increased and then decreased as the V/III ratio increased; the FWHM tended to decrease and then increase, also showing a turning point at the V/III ratio of 120. These results indicate that a V/III ratio of 120 is the optimum ratio for the growth of InAs QDs.

**
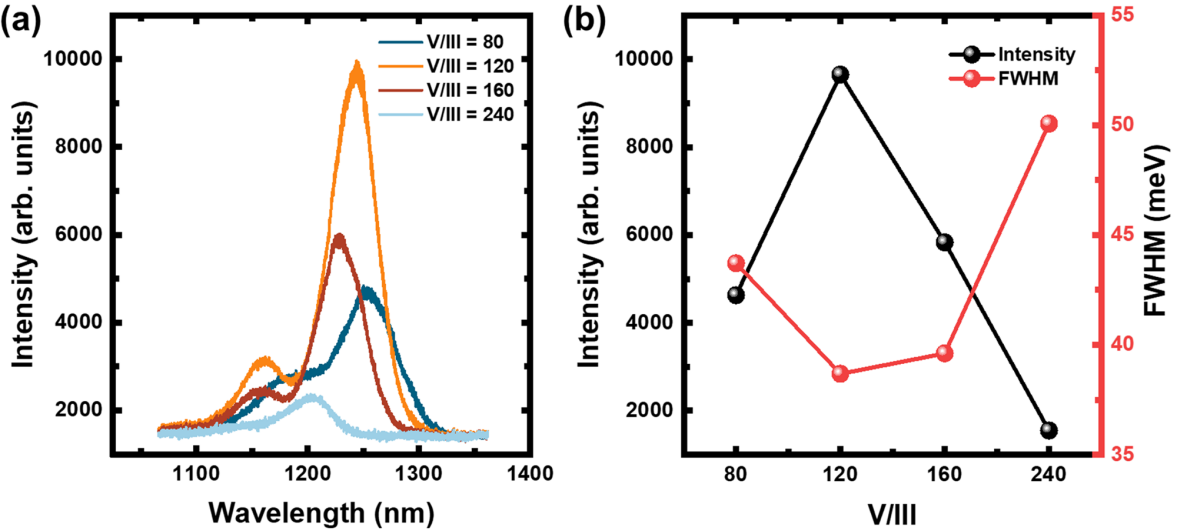
**

**Figure S1.** (a) The PL results. (b) The statistical results of the FWHM and intensity of PL results.

1. **The typical AFM results of QDs grown at different temperatures**

The typical AFM result of QDs grown at different temperatures are shown in **Figure S2**. As the growth temperature increases, the QD density decreases from 8.6 × 10^10^ cm^-2^ to 1.4 × 10^10^ cm^-2^.


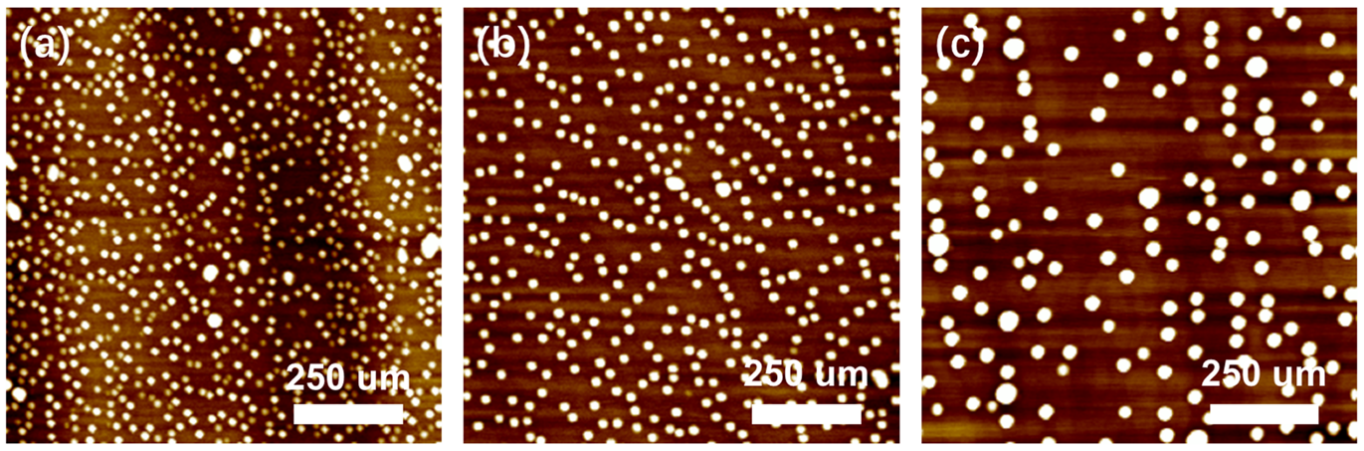


**Figure S2.** The typical AFM result of QDs grown at (a) low, (b) suitable and (c) high temperatures.

1. **The PL result of InAs QDs growth with different deposition amounts**

4 InAs/GaAs QD samples were grown, differing only in the amount of InAs deposited. The PL of the 4 samples were obtained with a laser power density of 423.25 W/cm², a higher excitation than others due to their weak emission. The results in **Figure S3a** indicate that intensity reaches its maximum at a deposition amount ratio of 2.6 monolayers (ML). The graph in **Figure S3b** illustrates the relationship between deposition amount and PL intensity, indicating that the PL intensity gradually increases as the deposition amount increases from 2.0 ML to 2.6 ML. However, the PL intensity decreases from 2.6 ML to 2.9 ML, indicating that 2.6 ML is the optimal deposition amount.


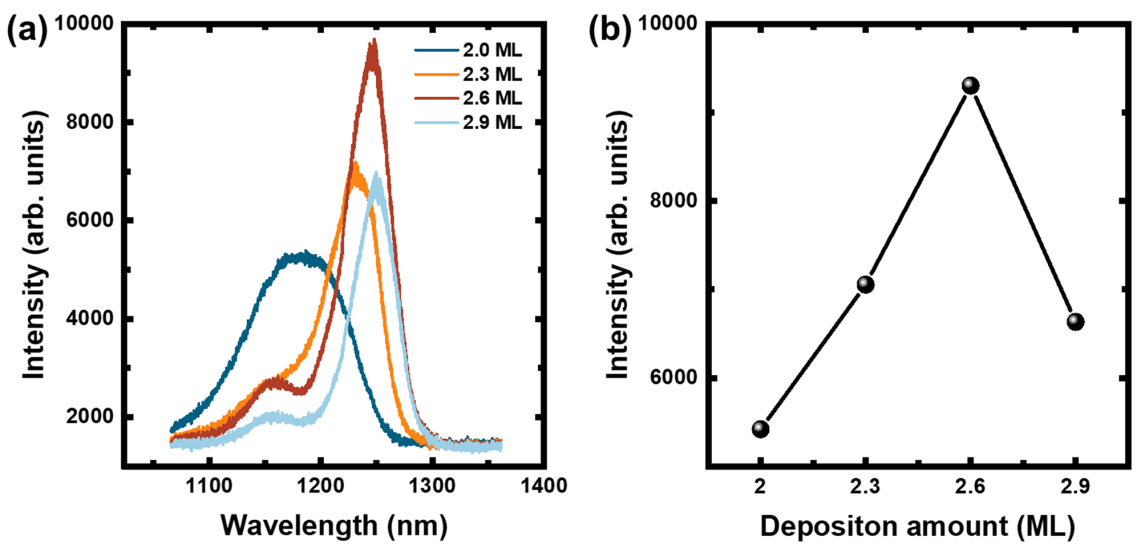


**Figure S3.** (a) The PL results. (b) The statistical results of the FWHM and intensity of PL results.

1. **The typical RHEED image with and without faintly discernible chevron streaks**


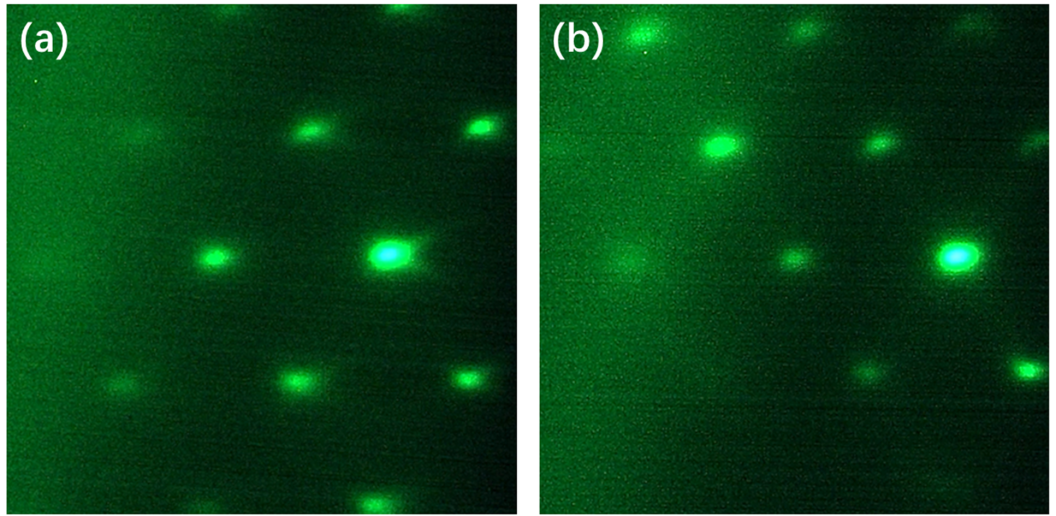


**Figure S4.** The typical RHEED image (a) with and (b) without faintly discernible chevron streaks.

1. **The details on modules in the GLAM block**

The local channel attention (LCA) module aims to enhance feature representations by emphasizing the relationships between different channels in the input feature maps, as shown in **Figure S5a**.^[1-4]^ This module employs a series of fully connected layers to calculate an attention vector showing each channel’s importance. The attention mechanism employs global average pooling to gather spatial information and then applies a convolution operation to generate attention coefficients for each channel. The output is generated by scaling the input features with these coefficients, allowing the model to highlight important channels while suppressing less informative ones. This approach facilitates improved representational learning and enhances the network’s ability to capture crucial features.

The local spatial attention (LSA) module enhances the attention mechanism by focusing on spatial dimensions within the feature maps, as shown in **Figure S5b**.^[2, 5-6]^ The process begins with a 1×1 convolution to reduce the number of channels. It then employs dilated convolutions with various kernel sizes to capture multi-scale spatial information. The resulting feature maps are concatenated and normalized using batch normalization. A second 1×1 convolution restores the original channel dimension. The spatial attention map is then upscaled to match the input size, allowing the model to highlight important spatial regions while preserving contextual integrity. By integrating both channel and spatial attention, this module significantly improves the model’s ability to understand context in feature representations.

**
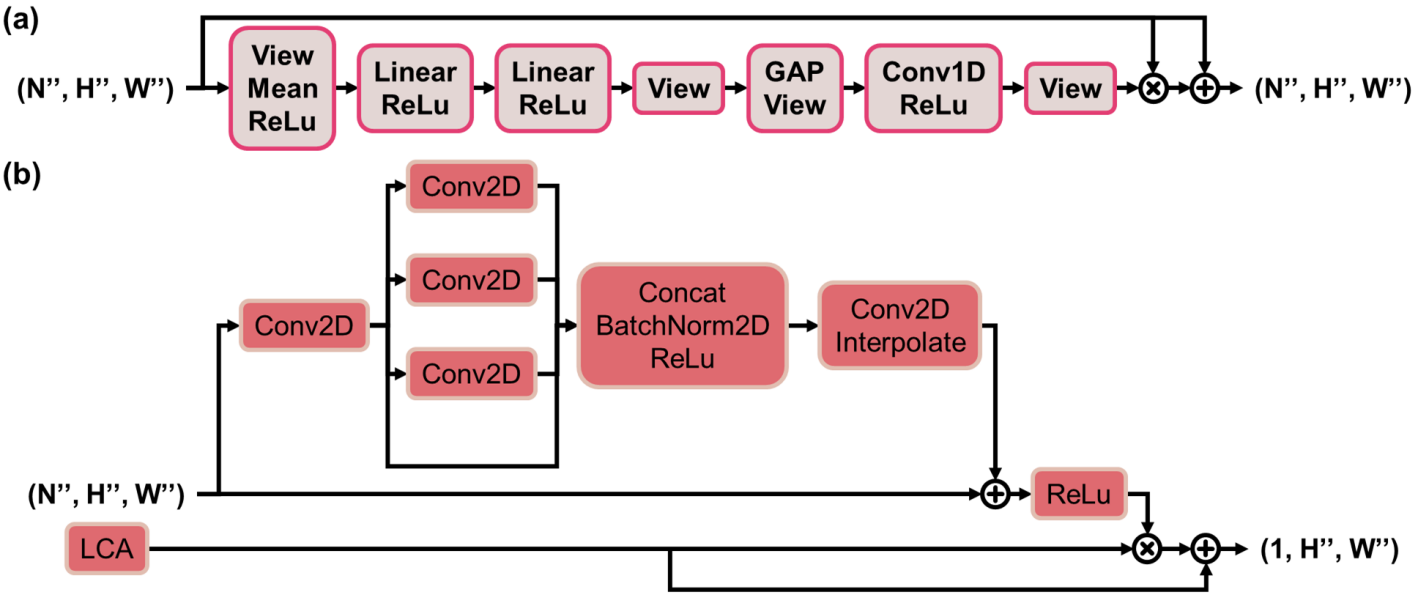
**

**Figure S5.** The structure of (a) LCA and (b) LSA.

The global channel attention (GCA) module improves the representation of feature maps by focusing on interactions between channels using a global context mechanism, as shown in **Figure S6a**.^[2, 7-9]^ Initially, the module employs global average pooling to gather spatial information, resulting in a more concise representation of the input feature maps. Next, two 1×1 convolutional layers generate query and key representations, which are combined through a bilinear product to calculate channel attention scores. The resulting attention matrix, normalized using a softmax function, indicates the importance of each channel and is used to adjust the original input features. This process effectively amplifies the contributions of informative channels while diminishing the impact of less relevant ones, thus enhancing the model’s capability to capture complex feature dependencies.

The global spatial attention (GSA) module enhances spatial representations through multi-scale convolutional operations to calculate attention, as shown in **Figure S6b**.^[2, 7, 10-11]^ This module consists of three convolutional layers with different kernel sizes, which extract query, key, and value representations from the input feature maps. The attention mechanism reshapes the query and key outputs to compute an attention matrix through a bilinear product, capturing important spatial relationships. The resulting attention scores are then applied to the value representation, emphasizing crucial spatial features while perserving contextual information. Finally, a 1×1 convolution restores the original channel dimensions of the output. This spatial attention mechanism allows the model to dynamically focus on important regions within the feature maps, improving its performance in complex visual tasks.

**
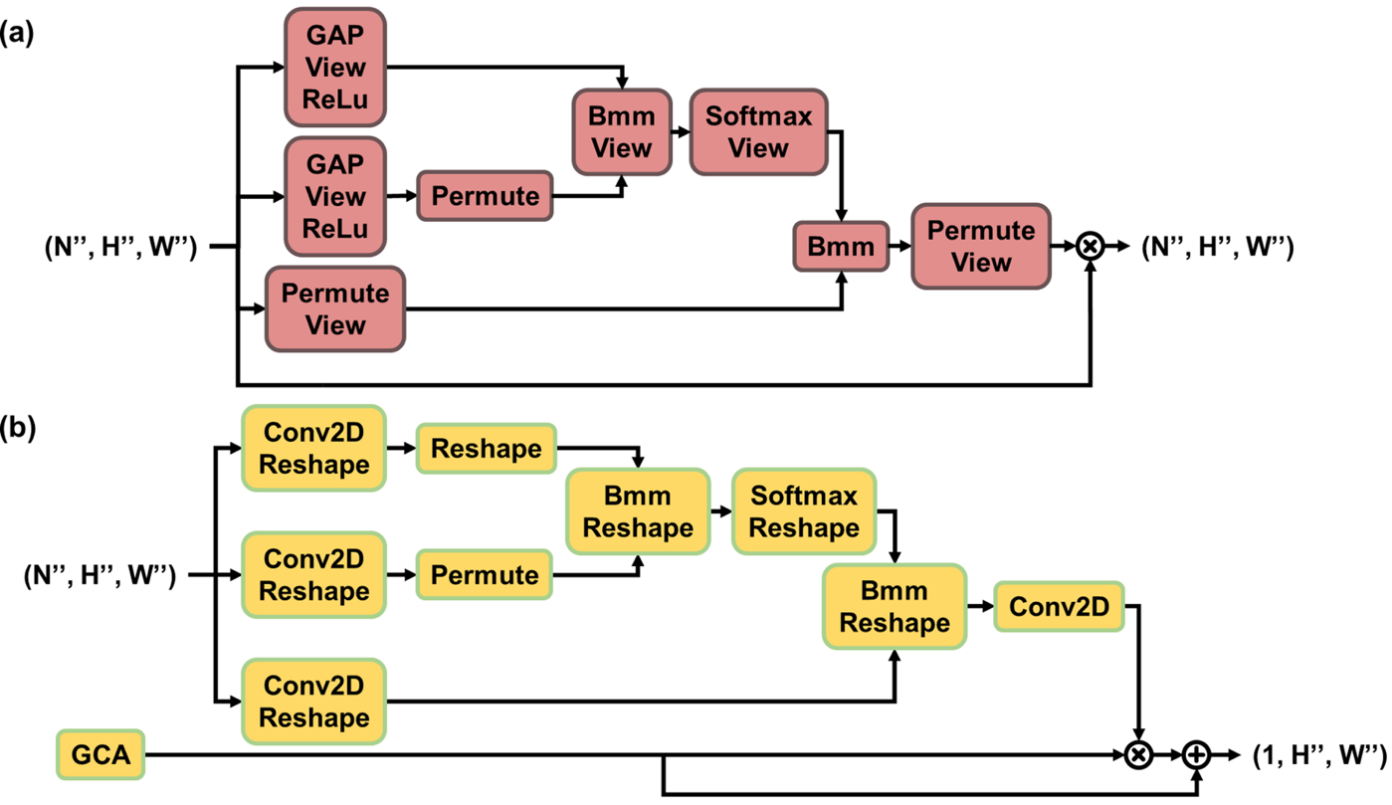
**

**Figure S6.** The structure of (a) GCA and (b) GSA.

1. **The training and validation speed of the two models**

To compare the training and validation speeds, we trained the “Shutter model” and the “Temperature model” using different structures and varying sample sizes as inputs, as shown in **Figure S7**. The Figure shows that as the training period increases, both the model’s training and validation accuracy tend to improve.


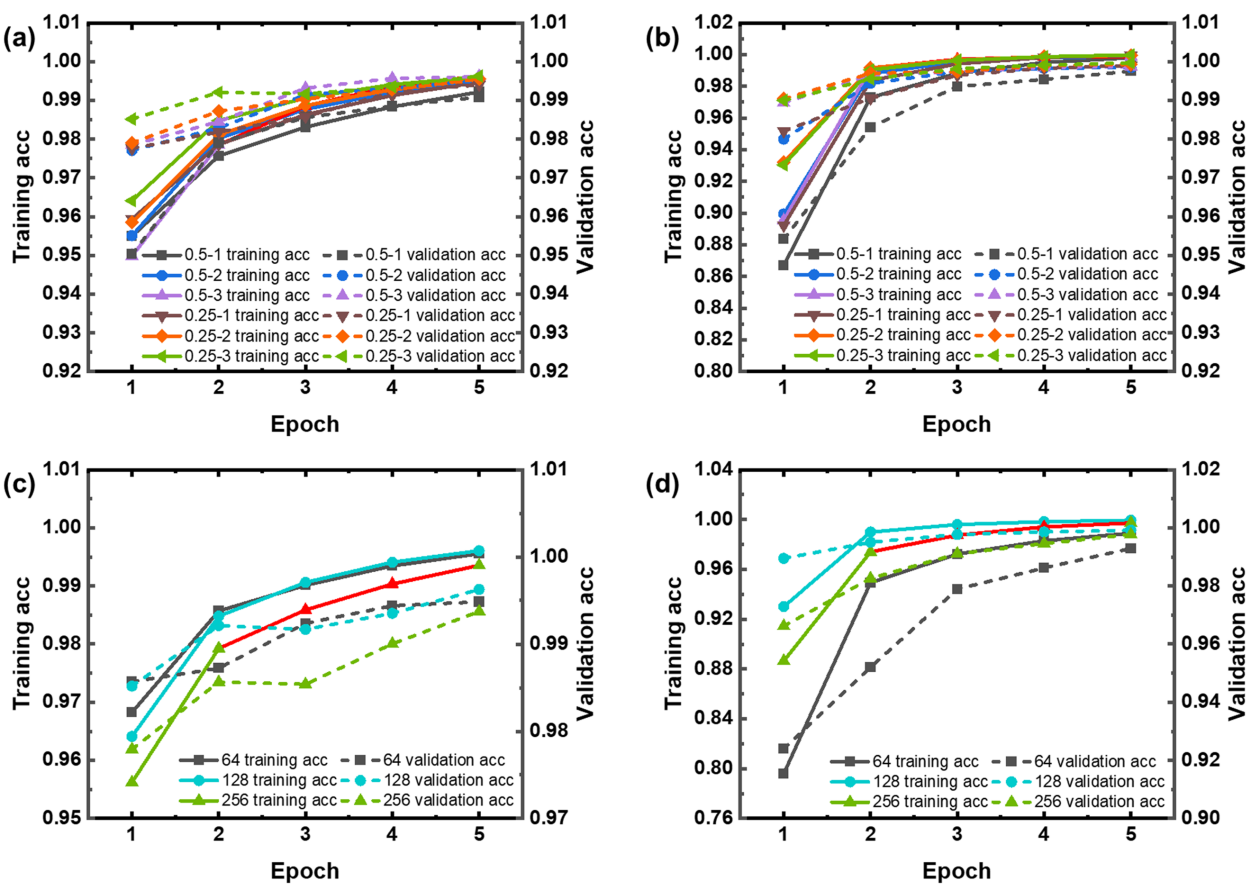


**Figure S7.** The training and validation process of (a) the “Shutter model” and (b) the “Temperature model” under different model structures. The training and validation process of (c) the “Shutter model” and (d) the “Temperature model” under different input sizes.

We thoroughly analyzed the data processing speeds for both the “Temperature model” and “Shutter model”, as shown in **Figure S8**. The results show that both models consistently maintain high efficiency, achieving a minimum processing speed of 100 samples per second, regardless of structural changes, as shown in **Figure S8a-S8b**. Furthermore, when comparing processing speeds for different input sizes, we observed a gradual decrease in speed with larger input sizes, as shown in Figure S8c-S8d. Nevertheless, both models still manage to achieve speeds close to 100 samples per second, demonstrating their scalability.


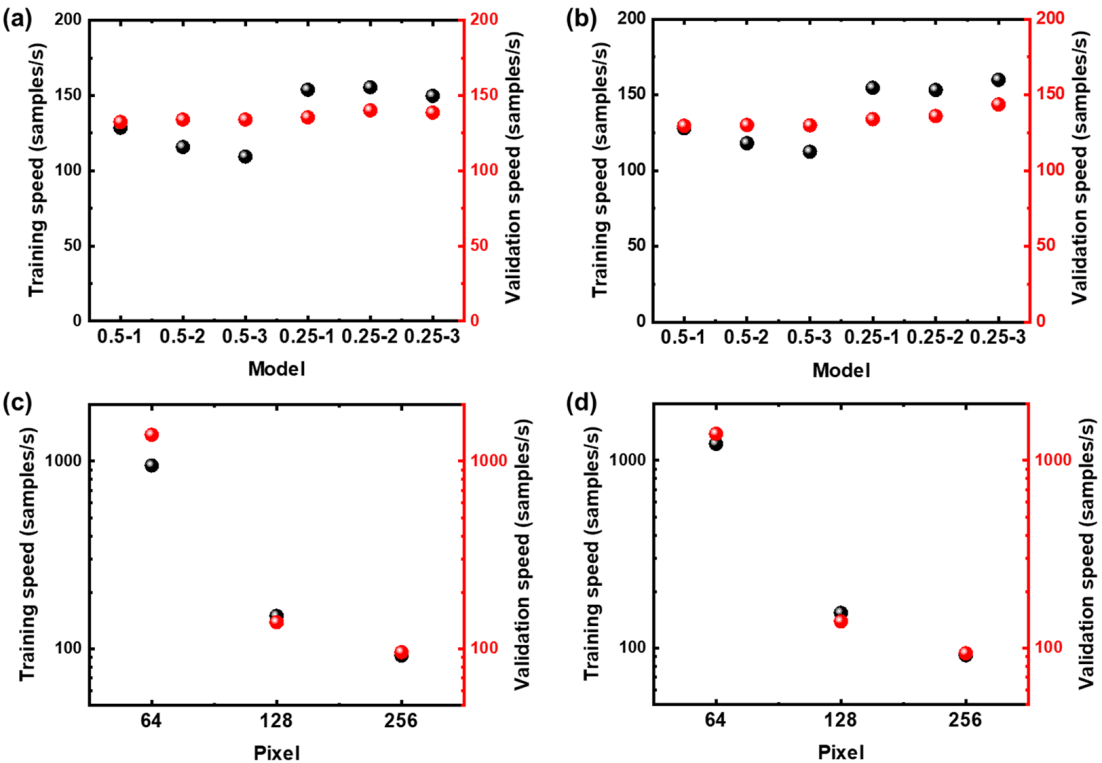


**Figure S8.** Speed of training and validation of (a) the “Shutter model” and (b) the “Temperature model” under different model structures. Speed of training and validation of (c) the “Shutter model” and (d) the “Temperature model” under different input sizes.

1. **The experiment setup and program interface**

The experiment setup and program interface are illustrated in **Figure S9-S10**. The left monitor displays the program's visualization interface. At the bottom of the monitor, the current voltage and controller for the RHEED power supply can be seen. Inside the cabinet, there are several temperature controllers. The temperature for the Ga cell is in the first position of the third row, while the temperatures for the In cell and substrate are in the second and third positions of the fourth row, respectively. Below the temperature controllers, there are shutter controls for all cells and the main shutter. In the bottom right corner, the manual shutter for the RHEED fluorescent screen is shown, which needs to be adjusted manually by a grower.


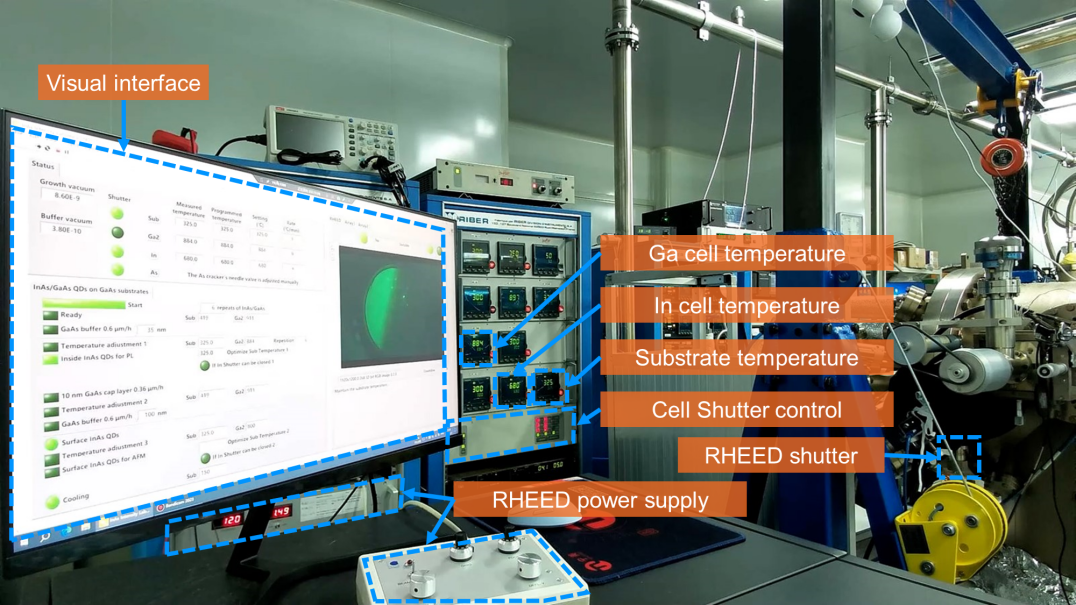


**Figure S9.** The experiment setup.

**
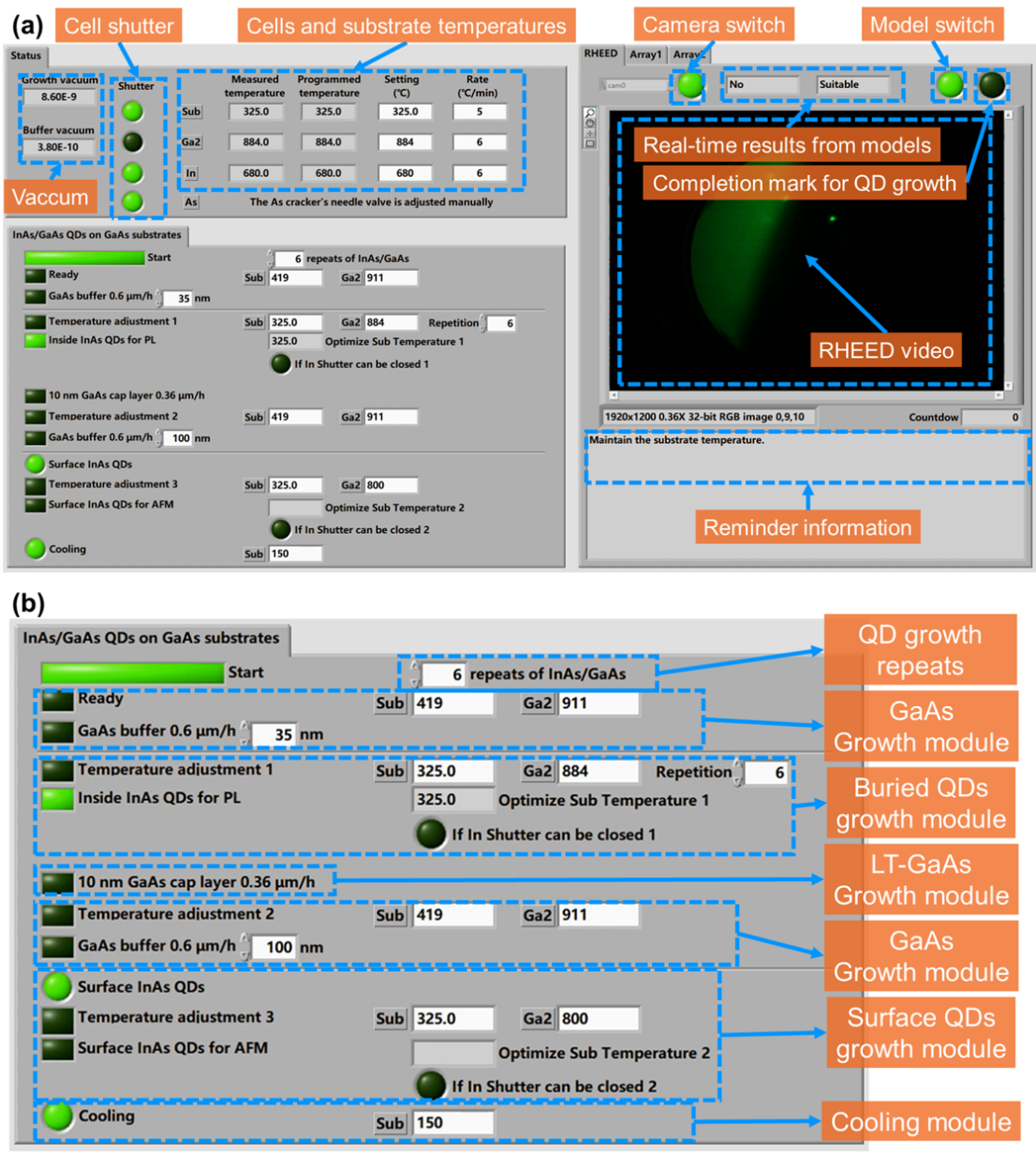
**

**Figure S10.** (a) The interfaces of all program components. (b) The “InAs/GaAs QDs on GaAs substrate” growth module.

1. **Details on setting the substrate temperature ramp rate**

During model deployment, it is essential to manage the temperature ramp rate carefully to avoid issues related to temperature overshoot. A rapid temperature change can cause fluctuations in the growth temperature of InAs QDs, while a slower rate may fail to reach the optimal growth temperature, resulting in experimental failure. The program addresses this by combining fast and slow temperature ramp rates. Initially, it optimizes the substrate temperature near the optimal growth temperature using a rapid rate of 30 °C per minute. Then, it employed a lower rate of 5 °C per minute to fine-tune the substrate temperature. The initial rapid temperature change caused an unstable growth temperature, which negatively impacted QD formation and the spectral performance of the QDs. Once QD growth in the first deployment is complete, the program will apply high-temperature annealing to remove the QDs from the surface before re-growing GaAs, ensuring a smoother sample surface.^[12-13]^ This adjustment ensured that QDs are grown at a suitable temperature, enabling the immediate growth of low-temperature (LT)-GaAs following the QD growth. Additionally, surface InAs QDs were prepared to investigate their distribution, density, and uniformity.

1. **The AFM and PL results of control sample A**

The control sample A was grown on the n-GaAs substrate. Single-layer InAs QDs were grown at a substrate temperature of 490 °C. Additionally, surface QDs were also grown to examine their morphology. As shown in **Figure S11a**, the QD density on the sample’s surface reaches 1.7 × 10^10^ cm^-2^, with noticeable variation in QD size. The PL of this sample is displayed in **Figure S11b**, showing an intensity of 1900.6 with a FWHM of 53.30 meV.


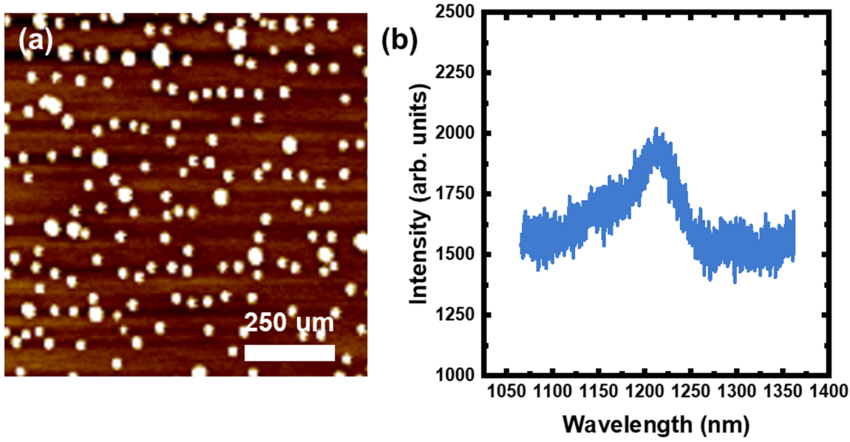


**Figure S11.** (a) The 1 μm × 1 μm AFM image. (b) The PL result.

1. **The AFM and PL results of control sample B**

The control sample B was also grown on the n-GaAs substrate with. Single-layer InAs QDs were grown at a substrate temperature of 475 °C following multiple optimizations of the growth conditions. Additionally, surface QDs were also grown to examine their morphology. As shown in **Figure S12a**, the QD density on the sample’s surface reaches 2.9 × 10^10^ cm^-2^. Notably, the larger-sized QDs show a significant reduction in number compared to control sample A. The PL of this sample is displayed in **Figure S12b**, showing an intensity of 4409.7 with a FWHM of 36.69 meV.


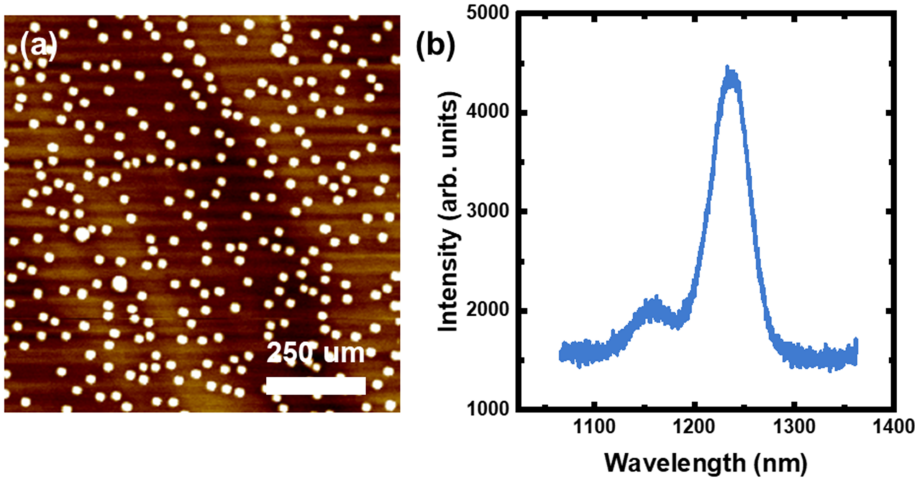


**Figure S12.** (a) The 1 μm × 1 μm AFM image. (b) The PL result.

1. **The data of single-layer buried QDs grown assisted by ML model**

**Figure S13** shows the data from single-layer buried QDs with the initial growth temperature the same as control sample A, prepared using the ResNet-GLAM model and controlled by the LabVIEW program. When the model was initially deployed, there was a temperature change of 20 °C in the substrate. This indicated that the initial temperature was not optimal for growing QDs with excellent optical performance, as shown in **Figure S13a**. As a result, the first growth of InAs QDs was removed through thermal annealing to prevent any deterioration in the optical performance of the sample.^[12-13]^ Subsequent model deployments showed a gradual decrease in temperature changes, suggesting that the model was approaching the optimal temperature for QD growth.

The growth time for each InAs QD is determined by measuring the period from the start of the growth process until the In shutter is closed, corresponding to the time between the initial “No” label and the “Yes” label in the “Shutter model”, as shown in **Figure S13b**. The growth time demonstrates minimal fluctuations during each InAs QD growth, indicating a stable deposition of InAs. This suggests that the QDs consistently completed their growth and achieved optimal optical performance within a stable deposition amount. It demonstrates high stability and reproducibility in sample growth. Additionally, the analysis of the number of labels produced during each deployment revealed a trend related to changes in substrate temperature. As shown in **Figure S13c**, during the initial deployment of the “Temperature model”, the “High” label was significantly more probable than the others, indicating that the initial growth temperature of the InAs QDs was too high. Subsequent deployments showed a gradual increase in the probability of the “Suitable” label, alongside a decrease in the “High” and “Low” labels. This trend suggests that the QD growth temperature became more suitable, leading to improved optical performance.

We captured typical RHEED images during the initial stages of QD growth for each model deployment. During the first deployment, we observed a clear ×4 reconstruction feature, while the ×2 reconstruction feature was evident during the second and third deployments, as shown in **Figure S13d-S13f**. This confirms that the initial growth temperature of InAs QDs was too high, while the temperatures during later deployments-after model guidance and LabVIEW adjustments- were more appropriate. Additionally, RHEED images captured after InAs QD growth for each model deployment are shown in **Figure S13g-S13i**. These images reveal a faintly discernible chevron streak feature surrounding the specular spot, demonstrating that the model accurately identified the optimal time for completing QD growth.

After the growth process, the samples were tested by *ex-situ* AFM and room-temperature PL. The 1 μm × 1 μm AFM image showed a uniform distribution of QDs with a density of 4.3 × 10^10^ cm^-2^, as shown in **Figure S13j**. The corresponding PL intensity reached 14066 with a FWHM of 28.17 meV, as shown in **Figure S13k**.


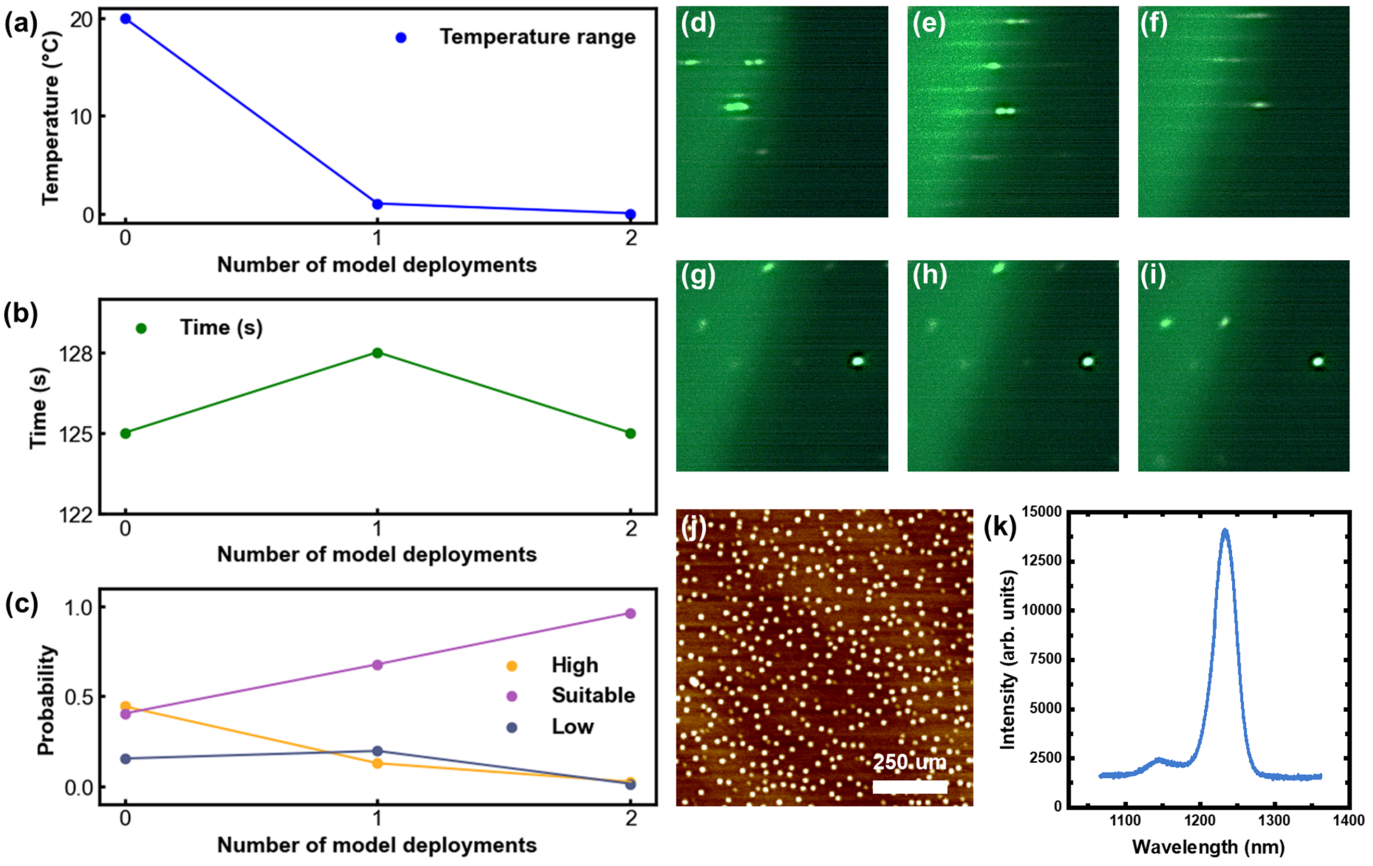


**Figure S13.** Data of single-layer buried QDs grown assisted by ML model: (a-c) Substrate temperature, growth time, and the output probability of the “Temperature model” during InAs QDs growth. (d-i) Representative RHEED images captured at the initial and near-completion stages of growth during each model deployment. (j) A 1 µm × 1 µm AFM image. (k) PL spectra of the sample.

1. **The AFM and PL results of control sample C**

A control sample C of 5-layer InAs QDs was further prepared using a conventional method based on the growth conditions of control sample B. The surface QD density of the sample reached 3.6 × 10^10^ cm^-2^, as shown in **Figure S14a**. The corresponding PL intensity reached 4310.3, with a FWHM of 36.24 meV, as shown in **Figure S14b**. These results indicate that the samples prepared using this method exhibit excellent performance, which can be used for a device structure.


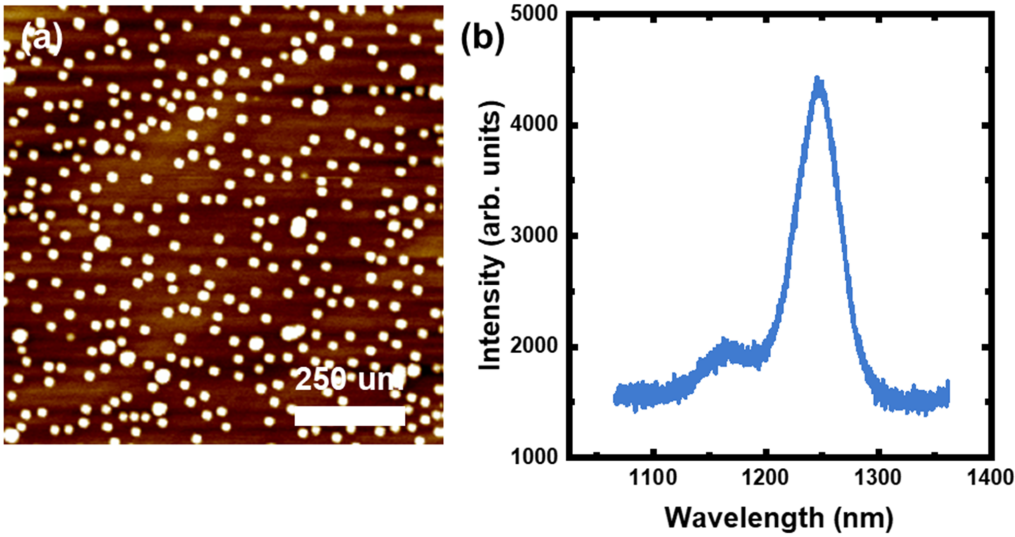


**Figure S14.** (a) The 1 μm × 1 μm AFM image. (b) The PL result.

1. **The TEM images of 5-layer InAs QDs**


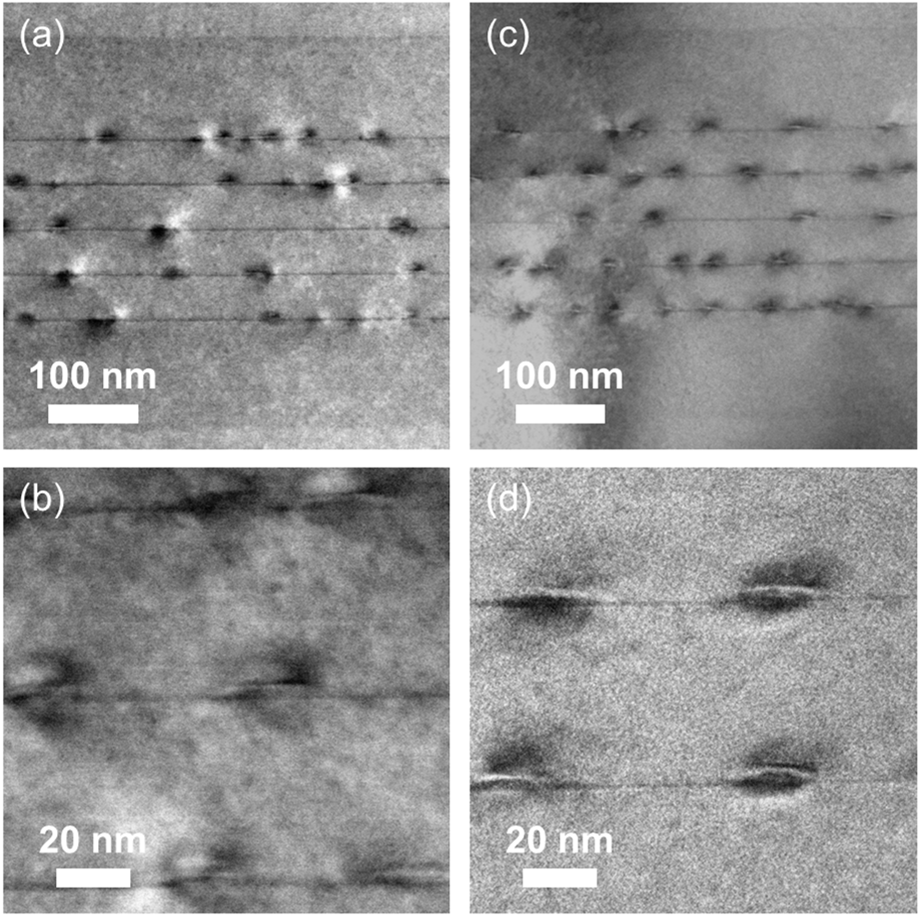


**Figure S15.** (a)(c) low magnification and (b)(d) high magnification.

1. **The two-dimensional and three-dimensional near-field intensity profile**

**
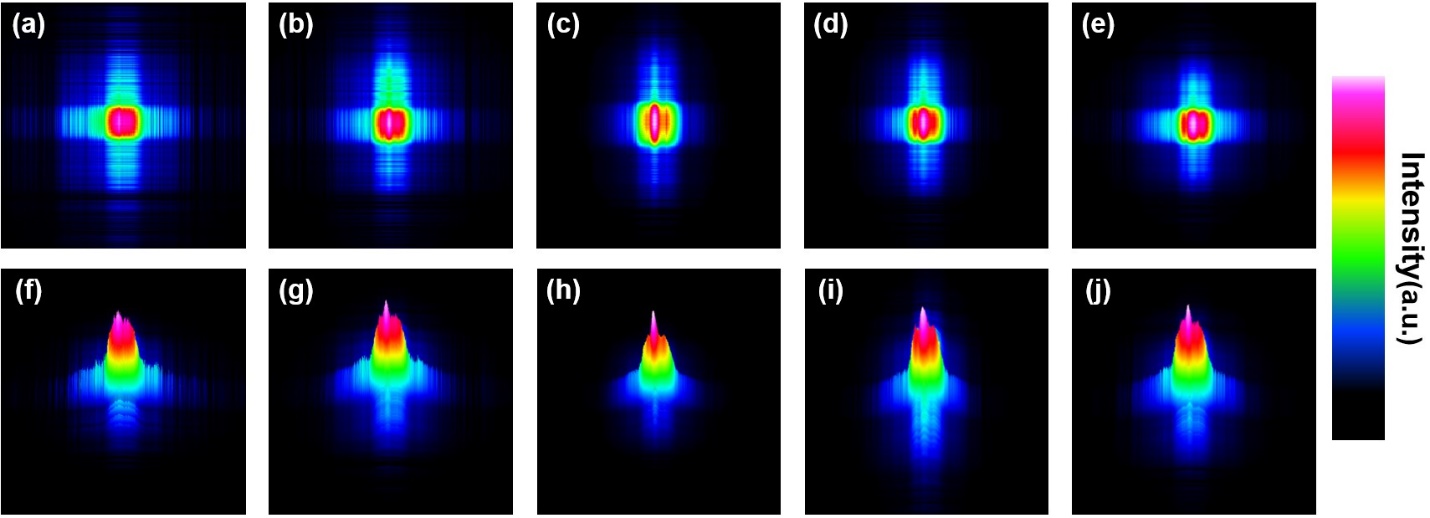
**

**Figure S16.** Two-dimensional (a-e) and three-dimensional (f-j) near-field intensity profile under different current density injections: (a) (f) 160 A/cm²; (b) (g) 260 A/cm²; (c) (h) 360 A/cm²; (d) (i) 460 A/cm²; (e) (j) 560 A/cm²

**References**

[1] Wang, Q., Wu, B., Zhu, P., Li, P., Zuo, W., Hu, Q., 2020, Proceedings of the IEEE/CVF conference on computer vision and pattern recognition, 11534.

[2] Song, C. H., Han, H. J., Avrithis, Y., 2022, Proceedings of the IEEE/CVF winter conference on applications of computer vision, 2754.

[3] Vosco, N., Shenkler, A., Grobman, M., 2021, Proceedings of the IEEE/CVF International Conference on Computer Vision, 345.

[4] Wan, D., Lu, R., Shen, S., Xu, T., Lang, X., Ren, Z., Mixed local channel attention for object detection, 2023*,* 123, Engineering Applications of Artificial Intelligence, 123, 106442, https://doi.org/https://doi.org/10.1016/j.engappai.2023.106442.

[5] Szegedy, C., Liu, W., Jia, Y., Sermanet, P., Reed, S., Anguelov, D., Erhan, D., Vanhoucke, V., Rabinovich, A., 2015, Proceedings of the IEEE conference on computer vision and pattern recognition, 1.

[6] Jin, L., Shu, X., Li, K., Li, Z., Qi, G.-J., Tang, J., Deep ordinal hashing with spatial attention, 2018*,* 28, IEEE Transactions on Image Processing, 28, 2173.

[7] Wang, X., Girshick, R., Gupta, A., He, K., 2018, Proceedings of the IEEE conference on computer vision and pattern recognition, 7794.

[8] Qin, Z., Zhang, P., Wu, F., Li, X., 2021, Proceedings of the IEEE/CVF international conference on computer vision, 783.

[9] Liu, Y., Shao, Z., Hoffmann, N., Global attention mechanism: Retain information to enhance channel-spatial interactions, 2021, arXiv preprint arXiv:.05561.

[10] Vaswani, A., Attention is all you need, 2017, Advances in Neural Information Processing Systems, 30, I.

[11] Chen, W., Ouyang, S., Tong, W., Li, X., Zheng, X., Wang, L., GCSANet: A Global Context Spatial Attention Deep Learning Network for Remote Sensing Scene Classification, 2022*,* 15, IEEE Journal of Selected Topics in Applied Earth Observations and Remote Sensing, 15, 1150, https://doi.org/10.1109/JSTARS.2022.3141826.

[12] Hsu, T. M., Lan, Y. S., Chang, W. H., Yeh, N. T., Chyi, J. I., Tuning the energy levels of self-assembled InAs quantum dots by rapid thermal annealing, 2000*,* 76, Applied Physics Letters, 76, 691, https://doi.org/10.1063/1.125863.

[13] Park, H. J., Kim, J. H., Yoon, J. J., Son, J. S., Lee, D. Y., Ryu, H. H., Jeon, M., Leem, J. Y., Step annealing effects on the structural and optical properties of InAs quantum dots grown on GaAs, 2007*,* 300, Journal of Crystal Growth, 300, 319, https://doi.org/https://doi.org/10.1016/j.jcrysgro.2006.11.337.
